# Emergence of the brain-border immune niches and their contribution to the development of neurodegenerative diseases

**DOI:** 10.3389/fimmu.2024.1380063

**Published:** 2024-05-28

**Authors:** Li Yang Tan, Grace Cunliffe, Michael Patrick Hogan, Xin Yi Yeo, Chansik Oh, Bohwan Jin, Junmo Kang, Junho Park, Min-Soo Kwon, MinYoung Kim, Sangyong Jung

**Affiliations:** ^1^ Department of Psychological Medicine, Yong Loo Lin School of Medicine, National University of Singapore, Singapore, Singapore; ^2^ Division of Neuroscience, School of Biological Sciences, Faculty of Biology, Medicine and Health, The University of Manchester, Manchester, United Kingdom; ^3^ Department of Medical Science, College of Medicine, CHA University, Seongnam, Republic of Korea; ^4^ Department of Pharmacology, Research Institute for Basic Medical Science, School of Medicine, CHA University, Seongnam, Republic of Korea; ^5^ Rehabilitation and Regeneration Research Center, CHA University School of Medicine, Seongnam, Republic of Korea; ^6^ Department of Biomedical Science, CHA University School of Medicine, Seongnam, Republic of Korea; ^7^ Department of Rehabilitation Medicine, CHA Bundang Medical Center, CHA University, Seongnam, Republic of Korea

**Keywords:** brain-border, neuroimmune, neurodegeneration, Alzheimer’s disease, Parkinson’s disease, multiple sclerosis

## Abstract

Historically, the central nervous system (CNS) was regarded as ‘immune-privileged’, possessing its own distinct immune cell population. This immune privilege was thought to be established by a tight blood-brain barrier (BBB) and blood-cerebrospinal-fluid barrier (BCSFB), which prevented the crossing of peripheral immune cells and their secreted factors into the CNS parenchyma. However, recent studies have revealed the presence of peripheral immune cells in proximity to various brain-border niches such as the choroid plexus, cranial bone marrow (CBM), meninges, and perivascular spaces. Furthermore, emerging evidence suggests that peripheral immune cells may be able to infiltrate the brain through these sites and play significant roles in driving neuronal cell death and pathology progression in neurodegenerative disease. Thus, in this review, we explore how the brain-border immune niches may contribute to the pathogenesis of neurodegenerative disorders such as Alzheimer’s disease (AD), Parkinson’s disease (PD), and multiple sclerosis (MS). We then discuss several emerging options for harnessing the neuroimmune potential of these niches to improve the prognosis and treatment of these debilitative disorders using novel insights from recent studies.

## 1 Introduction

For over a century, a fundamental principle in neurological research has been the understanding that the central nervous system (CNS) functions as a highly impermeable and immune-privileged organ system. This concept originated from a series of groundbreaking discoveries by Elrich (1885) (1), Bield and Kraus (1898) (2), and Lewandowsky (1900) (3), who showed that arsinine dyes, cholic acids or sodium ferro-cyanide, when administered intravenously, penetrate all organs except the brain. In 1900, Lewandowsky proposed the concept of a blood-brain barrier (BBB) but was met with criticisms regarding the specificity of the injected substrates used, as the substrates were thought to bind to plasma proteins in the blood. However, further evidence for the presence of a BBB was presented in 1913, when Goldmann systemically injected the acidic dye trypan blue into several species, including dogs and monkeys, and found that the brain remained unstained, although the choroid plexuses were stained (4, 5). In contrast, the entire parenchyma was stained when trypan blue was injected directly into the brain ventricles. This led Goldmann to postulate that the CNS is segregated from the blood circulation by a very selective barrier and that the choroid plexus is the host of this barrier that modulates substance entry into the CNS (6). Goldmann further proposed that cerebrospinal fluid (CSF) was the agent of substance transport for the CNS (7).

The concept of an immune privilege in the brain began to gain prominence after the discovery of the physical BBB and blood-cerebrospinal-fluid barrier (BCSFB). In 1921, Japanese scientist Shirai observed that transplanted rat sarcoma cells proliferate when implanted in the mouse brain parenchyma, but not when implanted into the muscles or skin (8). Shortly after in 1923, Murphy and Sturm reported that tumoral growth was inhibited when mouse tumours were co-transplanted into the brain with homologous spleen tissue (9), suggesting that peripheral immune cells were not present within the brain. Around the same time, in 1920–1921, Spanish neuroscientist Rio-Hortega described the brain as containing specialised ramified phagocytes that can self-proliferate (10), indicating that the CNS evolved to have its own unique innate immune system. Nevertheless, the term ‘immune privilege’ was only officially coined in 1948, when Brazilian-British biologist Sir Peter Medawar reported the lack of immune response to skin allografts transplanted to the brain and anterior chamber eyes of rabbits (11). At this time, the consensus was that peripheral immune cells were ‘absolutely’ restricted from the brain parenchyma due to the presence of a tight BBB and BCSFB preventing peripheral immune cell infiltration into the CNS.

However, the perception of this ‘absolute’ barrier between the peripheral system and the CNS began to shift in the 2000s with the discovery that activated peripheral immune cells, such as T cells and B cells, successfully enter and closely localise with the brain parenchyma at the borders of the CNS (12). Meningeal γδ T cells have even been suggested to play pivotal roles in secreting factors to maintain normal neural functions such as synaptic plasticity (13). In addition, it was previously believed that microglial major histocompatibility complex II (MHC-II) was solely responsible for causing neuroinflammation seen in neurodegenerative disease. However, it is becoming increasingly apparent that the microglial MHC-II is not essential for neuroinflammation in various neurodegenerative conditions (14, 15). Moreover, peripheral immune cell types, such as border-associated macrophages (BAMs) at brain-border niches, and infiltrating dendritic cells from the CSF are known to express MHC-II and can migrate to cervical lymph nodes to initiate adaptive responses (14, 16). Collectively, these observations aid in redefining our current understanding of a ‘relative’ immune privilege within the CNS.

Although numerous excellent reviews regarding the brain-border immune niches have been published previously (17, 18), the precise functions of these niches in neurodegenerative diseases remain unknown. Therefore, in this review, we will provide an overview of the immune niches and their functions, before updating the current understanding of the roles of the different niches relating to the progression of Alzheimer’s disease (AD), Parkinson’s disease (PD) and multiple sclerosis (MS) by referencing recent articles. We will discuss how these findings may improve or influence the future direction of the diagnosis, prognosis, and therapy of these progressively debilitating diseases.

## 2 Brain-border immune niches

### 2.1 Choroid plexus

The choroid plexus is a highly vascularised tissue that lines the pia mater within each ventricle of the brain, functioning to separate CSF from the blood. The choroid plexus consists of tightly bound epithelial cells with their basolateral surface surrounding a network of fenestrated capillary cores and loose stromal tissues. The apical surfaces of these cells contact the CSF within the brain ventricles and facilitate CSF production and secretion of signalling factors. The choroid plexus epithelium accounts for most of the BCSFB through maintaining tight junctions between epithelial cells (19). Away from the choroid plexus, ependymal cells continuously line the rest of the epithelium on the remaining walls of the CSF cavities. However, unlike choroid plexus epithelial cells, ependymal cells are held more loosely by desmosomes, and thus do not restrict movement between the CSF cavity and the parenchyma (20).

The choroid plexus stroma is a heterogenous, and dynamic immune environment located between the choroid plexus epithelium and the capillaries. It consists primarily of arachnoid cells, reticular fibroblasts, pericytes, and smooth muscle cells (21). Resident lymphoid and myeloid populations such as CD4^+^ T helper cells (Th1, Th2, Tregs) (22), CXCR3^+^ dendritic cells, and macrophages (23), are sparsely distributed within the stroma, but still in close proximity to stromal elements. This unique apposition allows the choroid plexus to respond to antigen-presenting cells (APCs) or inflammation factors that may present either from the CSF or the systemic circulation.

**Figure 1:**
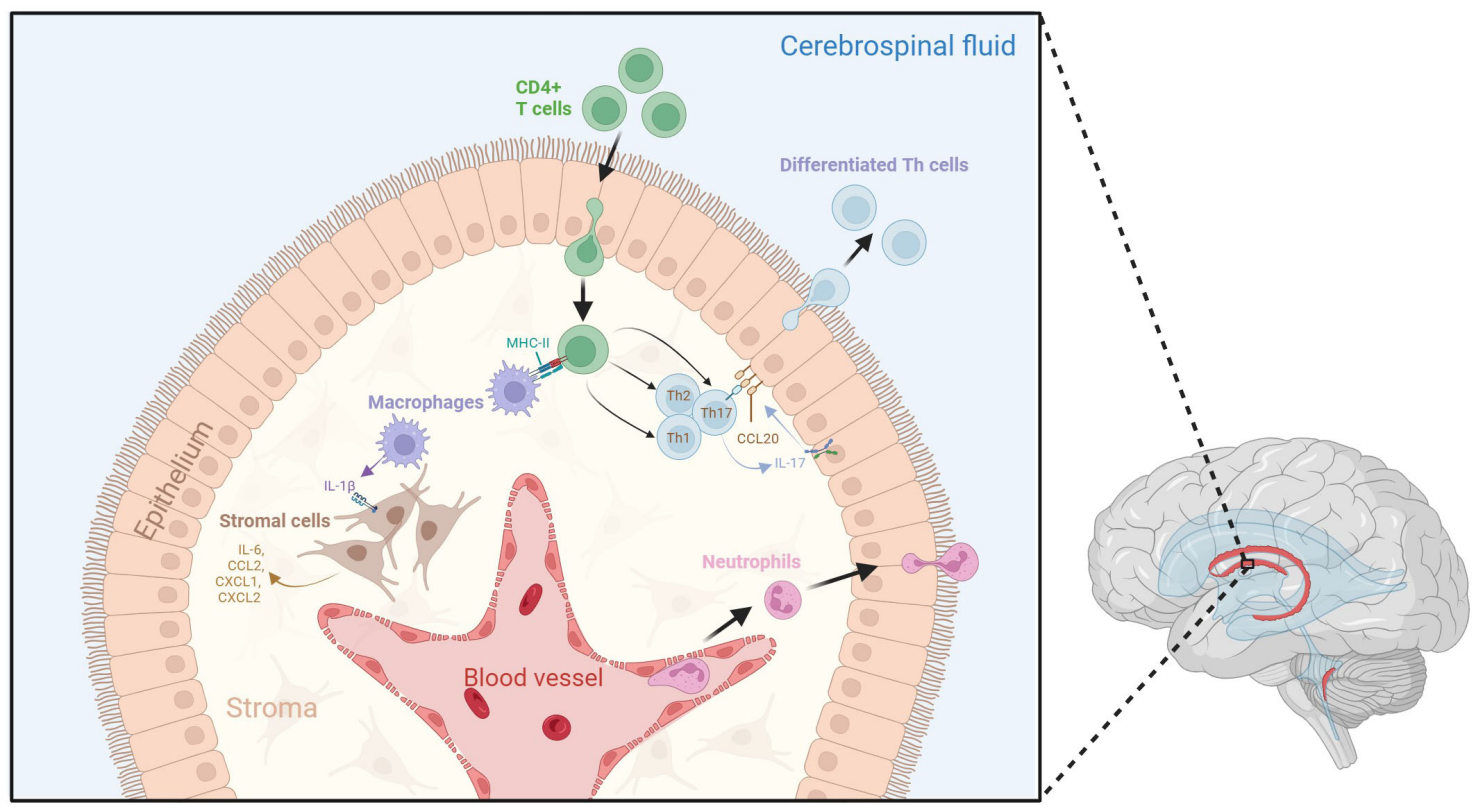
Dynamic neuroinflammatory responses at the choroid plexus. The choroid plexus is an active immune niche that monitors immune factors from the blood and the CSF. During the inflammatory response, surveilling CD4+ T cells infiltrate the choroid plexus and are presented with antigen-loaded MHC-II by resident macrophages. This triggers their differentiation into various T helper cell subtypes. Th17 cells secrete IL-17, which promotes choroid plexus epithelial CCL20 expression, therefore allowing for the adhesion of lymphocytes to the choroid plexus epithelium and aiding their transmigration into the CSF. Macrophages within the stroma secrete IL-1β due to interactions with activated CD4^+^ T cells. IL-1 β interacts with stromal cells, which in turn secrete pro-inflammatory cytokines and chemokines such as IL-6, CCL2, CXCL1, and CXCL2. Tight junctions between choroid plexus epithelial cells become compromised following inflammation, which may lead to increased movement of immune cells across the epithelium. Additionally, neutrophils from the blood stream accumulate in the choroid plexus stroma, allowing them to infiltrate the CSF. Figure created with BioRender.

Recent research has shed light on the complex immunological interactions within the choroid plexus stroma (**Figure 1**). In septic mouse models induced by intravenous lipopolysaccharide (LPS) administration, activated APCs such as M1 macrophages can infiltrate the choroid plexus and release interleukin (IL)-1β (21). Stromal cells expressing IL-1 receptor type 1 (IL-1R1) and IL-1 receptor accessory protein (IL-1RAcP) can respond to IL-1β and subsequently secrete proinflammatory factors such as IL-6, CCL2, CXCL1, and CXCL2 (21). Additionally, when stimulated by cytokines such as interferon-gamma (IFN-γ) and IL-17 from CD4^+^ Th cells, the choroid plexus epithelium expresses unique trafficking molecules and releases chemoattractant ligands such as CCL20, which promote the transmigration of preactivated B cells and T cells into the CSF in experimental autoimmune encephalomyelitis (EAE) mouse models for MS (24, 25). Neutrophils have also been shown to infiltrate the choroid plexus from the bloodstream following traumatic head injury, before accumulating in the CSF around the site of injury (26).

As individuals age, the composition of the choroid plexus stroma undergoes gradual changes due to local and peripheral senescence (27). The tight junctions of the choroid plexus epithelium are greatly compromised (28), and there is an increase in stromal leukocyte proportion, particularly T helper and cytotoxic T cells (29). Consequently, there is a shift in the cytokine profile, marked by elevated levels of inflammatory cytokines such as IL-1β, tumour necrosis factor-alpha (TNF-α), and IFN-γ (22, 27, 30), contributing to a progressively inflammatory brain.

### 2.2 Meninges

The meninges are a series of membranes covering the brain and spinal cord, forming a continuous barrier between the CNS and periphery. The primary roles of the meninges are to protect the CNS from trauma and to provide structural support for nerves, vasculatures and lymphatics that supply the CNS (31). Conventionally, the meninges are divided into three distinctive structural layers: the outermost dura mater, the arachnoid mater, and the innermost pia mater, which forms direct contact with the brain parenchyma. Fluid-filled cavities between the arachnoid and pia mater are termed subarachnoid spaces and those between the pia mater and parenchyma are called subpial spaces (32). The pia mater, subarachnoid space and arachnoid mater are collectively referred to as the leptomeninges (33).

The thickest and outermost lining of the meninges is known as the dura mater. This fibrous membrane covers the interior of the skull, and receives its own innervation and vascular supply at the apical surface from branches of the external carotid arteries (34). Histologically, the dura mater is further characterised into three major layers: the periosteal layer, which lines the inner surface of the cranium; the meningeal layer, which consists of a dense, fibrous membrane that lies underneath the periosteal layer; and the flattened dural border cell (DBC) layer which attaches to the arachnoid mater. Large cavities between the periosteal and meningeal layers are known as the dural venous sinuses. These provide an avenue for CSF to empty from the subarachnoid space through lymphatic channels into the internal jugular veins. In a healthy brain, there is no subdural space between the dura mater and the arachnoid mater (34).

The arachnoid mater is the middle layer of the meninges and comprises a thin layer of web-like connective tissue. The arachnoid mater is less densely vascularised compared to the dura mater and does not receive any innervation. However, the subarachnoid space that lies beneath the arachnoid mater contains CSF as well as vasculature, including arteries and veins. Lymphatic vessels have also been reported to reside adjacent to the subarachnoid space (35). Parts of the subarachnoid mater project into the dural venous sinuses as arachnoid granulations, allowing drainage of CSF from the subarachnoid space into the dural sinuses (36).

Though the dura and arachnoid mater can easily be separated from the parenchyma, the pia mater attaches strongly to the convoluted surface of the brain as the innermost layer of flattened cells, forming a thin fibrous tissue (37). Lacking tight junctions, the pia mater is permeable to small solutes such as urea (35), and is highly vascularised, with many vessels permeating the membrane into the brain (38). The pia mater shields the parenchyma from the CSF at the subarachnoid space and, at the cortex, coats the penetrating arterioles (but not venules) of the parenchyma (36). However, as vessels become smaller, the pial lining becomes more discontinuous (39), resulting in capillaries having direct contact with glia limitans. The fluid-filled cavities that form between the vessels and glia limitans of the brain parenchyma are called perivascular spaces, also known as Virchow-Robin spaces. These form the glia-lymphatic (glymphatic) system that drains most of the CSF from the subarachnoid space (40).

Recently, a fourth meningeal layer, defined as the subarachnoid lymphatic-like membrane (SLYM), has been proposed (41). Resembling mesothelium, this comprises only a sparse layer of Prox1^+^ lymphatic endothelial cells and loosely organised collagen fibres that encase pial vessels, separating the subarachnoid space into two distinct compartments; a superficial and a deep compartment (41, 42). Functionally, the SLYM restricts the passage of molecules smaller than 3 kDa, and therefore serves as a physical filter regulating material exchange between subarachnoid space compartments (41). Furthermore, it facilitates the direct exchange of small solutes between the CSF and venous blood, due to its proximity to the endothelial lining of the meningeal venous sinus (41). Notably, a significant accumulation of CD45^+^ cells has been observed near the pial vessels on the brain surface, and this increases following the induction of inflammation with LPS, as well as with ageing (41). Macrophages expressing LYVE1, CD206, and CD68, as well as CD11c^+^ dendritic cells have also been reported to reside in the SLYM (41). The presence of immune populations within this membrane may suggest that the SLYM is an active immune niche that functions as an assembly point for immune cells and regulates immune exchanges between the blood stream and the CSF between superficial and deep compartments of the subarachnoid space. Further investigation is necessary to understand the functional interplay of the SLYM with other meningeal layers, and its modulation of neuroinflammation in the brain parenchyma.

Another recent study unveiled multiple lymphoid-like structures within the dura of mice that closely associate with the infiltrating venous plexus (43). These tissues comprise a rich network of PV1^+^ fenestrated blood vessels and LYVE1^+^ lymphatic vessels, intertwined in a stroma of fibroblastic reticular cells. This network harbours germinal centre-like hubs, and contains CD11c^+^ myeloid cells, CD3^+^ T cells, B cells, neutrophils, macrophages, dendritic cells and (to varying degrees) their immature progenitors (43). The largest of these clusters was found mainly surrounding the rostral-rhinal confluence of the sinuses, located superior to the olfactory bulb (43). With their proximity to the cranium, these lymphoid tissues, especially those in the rostral-rhinal hubs, are in contact with small, ossified channels through a recess in the bone, and potentially derive their progenitor populations from the cranial bone marrow (CBM) (43). The same study suggested that, in addition to sampling CSF from within the dura, lymphoid hubs can detect antigens and mount responses to infections originating from the olfactory tract, CSF, and peripheral blood circulation. This is due to the presence of fenestrated blood vessels and the proximity of lymphoid hubs to the olfactory tract. When vesicular stomatitis virus (VSV) was introduced intranasally, the cytokine milieu and cellular compositions of dural-associated lymphoid tissues (DALTs) were altered, characterised by increased IL-12 expression and enhanced distribution and activation of B cells into plasma cells (43), thereby triggering a humoral response. Additionally, microbeads were observed to localise within these DALTs following their intravenous injection (43). This evidence indicates that the meningeal hubs actively contribute to preventing external infections from breaching the CNS.

**Figure 2:**
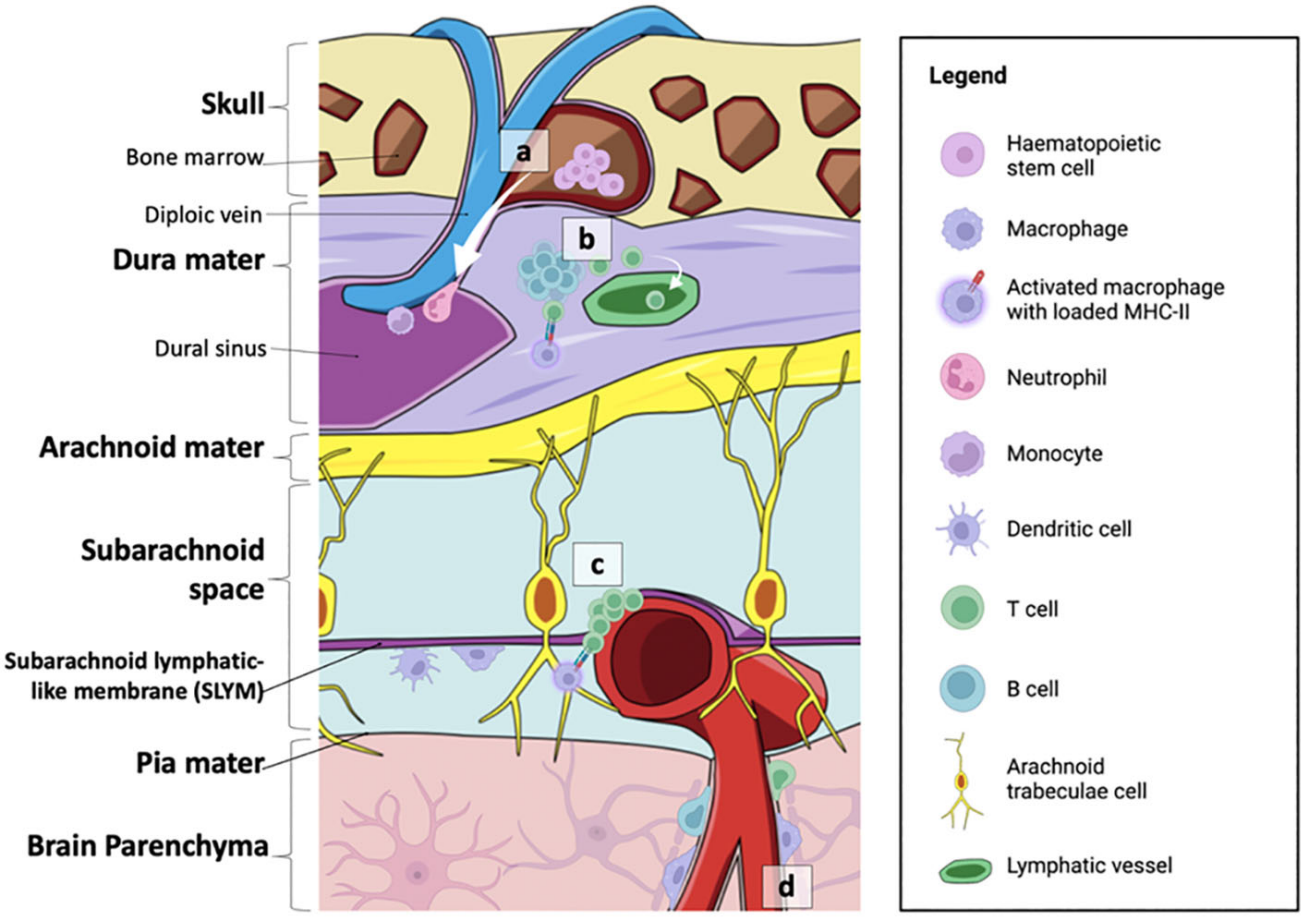
Summary of the neuroimmune niches within the skull-meninges-brain axis. **(A)** The cranial bone marrow (CBM) provides a source of haematopoietic stem cells, which supply lymphoid and myeloid cells to the dural sinuses during inflammatory events. **(B)** Germinal-like centres in the dural-associated lymphatic tissues (DALTs) contain lymphocytes. T cell populations in the meninges are most likely derived from the blood or CSF. T helper cells can be activated by MHC-II-presenting macrophages within the dura mater, and subsequently activate B cells and cytotoxic T cells. Immune cell activity within lymphoid hubs may help prevent the entry of external infection into the brain. **(C)** T cell clusters and MHC-II-presenting macrophages are also found at the subarachnoid lymphatic-like membrane (SLYM), closely associated with pial blood vessels. The SLYM may therefore serve as an active immune niche that regulates immune exchanges between the blood stream and the CSF. **(D)** Perivascular spaces house resident immune cells, such as perivascular macrophages, as well as a smaller population of B cells and T cells. Figure created with BioRender.

The composition of lymphoid hubs along the dura appears to change with increasing age and disease progression (44, 45). Interestingly, while B cells were found to be derived from the CBM, lineage tracing of the cells did not reveal an increase in CD4^+^ T cell populations in the CBM, suggesting that the enhanced localisation of CD4^+^ T cells within the meninges may be derived from systemic circulation (46). An overview of the immune niches within the skull-meninges-brain axis is shown in **Figure 2**.

### 2.3 Perivascular spaces

Perivascular spaces are fluid-filled cavities that lie between penetrating arterioles and the brain parenchyma. At the basal section of arterioles, perivascular spaces are continuous with the subarachnoid space (and therefore CSF) of the meninges and separated from the brain parenchyma by the pia mater (47). As vessels enter the cortex through the pia mater, the vascular endothelium and its basal membrane are in direct contact with the end feet of the glia limitans, forming the primary structure of the BBB (48). Perivascular spaces are established between tight junctions of the vascular endothelium and the astrocytic end feet and contain interstitial fluid from the outflow of blood from vessels, and CSF from the subarachnoid space. Although small in diameter, perivascular spaces together form an extensive network of glymphatic channels that function to drain CSF from the subarachnoid space (40) and remove neurometabolic waste from the brain, particularly during sleep (49, 50).

Pericytes and native innate immune cells are the primary cell types found within perivascular spaces. Pericytes play crucial roles in maintaining the structure of the BBB by regulating vascular development, angiogenesis, extending glial limitans end feet, and mediating inflammatory processes associated with immune cells. To fulfil this diverse range of functions, pericytes exhibit stem cell-like properties, enabling them to differentiate into angioblasts, neural progenitor cells, endothelial cells, and even microglia-like phenotypes (51, 52).

Regarding perivascular innate immune cells, those found at basal parts of the arterioles have been identified as BAMs (also called perivascular macrophages) positive for CD45, CD11b, CX3CR1, Ly6C and CD38, while CD119^+^ microglia are present in perivascular spaces of capillaries (53). Conversely, lymphocytes such as CD8^+^ tissue resident memory T cells and B cells are typically found in relatively low numbers in the perivascular spaces of the corpus callosum under normal physiological conditions (54), and are likely recruited to these spaces only during pathological states. Interestingly, these observations collectively suggest that perivascular spaces may harbour their own distinct population of self-proliferating immune cells (55).

The identification of perivascular spaces via magnetic resonance imaging (MRI) scans may serve as an important diagnostic and prognostic criterion. In both neonates and adult humans, perivascular spaces that are <2 mm in diameter are considered normal (47). Dilation of perivascular spaces has been associated with various conditions including old age (40), hypertension, vertigo, cysts (56), neuroinflammatory disorders such as dementia, MS (57), secondary PD, mega-encephalopathy, hydrocephalus (58), and autism spectrum disorder (ASD) (59). Currently, the cause of perivascular space dilation is not fully known but may be linked to obstruction of glymphatic flow. Interestingly, sleep (a processes commonly disrupted in old age and neurodegenerative disease) was recently reported to promote glymphatic clearance of metabolic waste by inducing rhythmic oscillations at delta (0.5–4 Hz), theta (6–10Hz), spindle (12–15Hz), and ripple (140–200Hz) wavelengths by synchronising neuronal activity (60). Moreover, aquaporin 4 (AQP4) proteins at astrocytic end feet also appear to have pivotal roles in regulating interstitial fluid pressure at the perivascular spaces (61). Additionally, arterial pulsation (62, 63), and peptide signalling (64), have been suggested to contribute to CSF dynamics and glymphatic clearance. Based on these findings, several hypotheses have been proposed surrounding the cause of perivascular space dilation, and include mechanical stress arising from impaired CSF pulsations (65, 66), increased vascular permeability (67), obstruction of downstream lymphatic drainage, and atrophy of the parenchyma (68, 69), all of which may lead to increased fluid exudation into the perivascular space. In hypertensive rat models, microglia that release inflammatory cytokine IL-1β have been observed to trigger the overexpression of prostaglandin E2 (PGE2) (70) and affect pericyte-endothelial cell interactions, resulting in vascular destabilisation and increased vascular permeability (71). However, the precise extent of immune cell involvement in the dilation of perivascular spaces is not clear.

### 2.4 Cranial bone marrow

Bone marrows are immune reservoirs for cells of various haematopoietic lineages. In normal physiology, the bone marrows house and deliver cells from myeloid and lymphoid lineages to the peripheral blood and surrounding tissues (72). The CBM is no exception; it has a distinct molecular composition and provides a direct source of immune cells to the CNS, thus shaping neuroimmune responses (73). The proximity of the CBM to the brain allows for the penetration of diploic veins from the meninges into the cranial marrow cavities through small micro-osseous channels, particularly at the frontal, parietal, and occipital cranium (74). These channels establish a route for direct communication between the CBM and the CSF, facilitating the exchange of immune cells, signalling molecules, or even pathogens (75), that may exacerbate wider immune responses.

In healthy mice, the CBM is responsible for maintaining the meningeal lymphocyte populations. For example, early B cells have been suggested to migrate from the CBM through miniature skull-meninges channels into the dural stroma (46), likely settling within DALTs (43). The lack of adaptive immune reactivity in the CBM suggests that clonal selection and full development of B cells could occur within the germinal-like centres in DALTs (43, 46), although this is not yet certain. Interestingly, cells that migrated from the CBM to the dura were not reported to express any T cell markers, suggesting that T cell localisation within the meninges is derived either from the blood or from the CSF (76). This indicates that blood-based or meningeal-based tracing is somewhat specific to lymphocyte populations at the meninges, and that T cells may be important for the prognosis of neurological diseases that arise from peripheral inflammation.

Recently, several groups have attempted to shed light on the conditions required to promote haematopoietic stem cell differentiation and migration from the CBM. CSF has been suggested to transport CSF-derived factors through skull meningeal channels of the cranium (77). This process activates signalling pathways crucial for inducing myelopoiesis and migration to the underlying dura (77). In EAE mice, induction of the CXCL12-CXCL4 axis was shown to promote T cell activation and migration into the CBM, leading to myeloid differentiation of haematopoietic stem cells into Ly6b^+^ or Ly6C^+^ macrophages and neutrophils that subsequently invade the CNS (78).

Additionally, the CBM composition appears to change with age (73, 79). Single-cell sequencing has revealed upregulation of senescence markers in mesenchymal cells within cranial stem cell niches and immune cells closely associated with inflammation (79). In adult humans, imaging techniques such as MRI have been utilised to correlate age-related changes with alterations in bone marrow composition. Analysis of apparent diffusion coefficient (ADC) values from MRI scans of five hundred subjects demonstrated significant alterations in the parietal and occipital bone marrow with increasing age (80), indicating changes to cell density (81–83). These inflammatory and age-related changes suggest a potential link between the CBM composition and brain health, although further research is required to determine the relationship between these alterations and age-related neurodegenerative diseases.

### 2.5 Brain-border lymphatics

In most peripheral tissues, interstitial fluid (ISF) is drained from organs by perforating lymphatic vessels. The lymphatic system also functions as an active immune surveillance system, with downstream lymph nodes housing germinal centres for pathogen recognition, and endothelial cells that secrete specific cytokines and express membrane adhesive factors for leukocytic diapedesis into the surrounding tissues (84). Although the CNS does not possess perforating lymphatics, recent advancements in high-resolution imaging techniques have revealed the presence of lymphatic channels coursing in a specific manner within the dura mater of transgenic Prox1-EGFP-expressing mice stained for LYVE1 (85). Additionally, leptomeningeal lymphatic endothelial cells (LLECs) have been suggested to reside in non-lumenised lymphatic endothelium within the leptomeninges (86). These brain-border lymphatic vessels and LLECs are now being increasingly recognised as crucial players in modulating CNS immunity, by facilitating the drainage of CSF from the meninges and clearance of metabolic waste products, whilst also serving a potential role in immune cell surveillance.

CSF from the brain parenchyma is generally known to flow through one of two different lymphatic routes. At the caudal end of the brain, CSF is drained from the sigmoid sinus through basolateral lymphatics at the dura, passing through the jugular foramen to lateral deep lymphatics, before directly arriving at the lateral cervical lymph nodes (85). In contrast, at the rostral parts of the brain, particularly the cavernous sinus and olfactory bulb, lymphatics pass through the cribriform plate to the olfactory epithelium adjacent to the olfactory neurons (87). From the olfactory epithelium, the fluids are drained into olfactory lymphatics which travel posteriorly to the nasopharynx into the deep medial lymph nodes (87).

A recent study has shown that, in both mice and macaques, the olfactory lymphatics link posteriorly to a lymphatic plexus superior to the nasopharynx bone at the skull base (85). This plexus structure, termed the nasopharyngeal lymphatic plexus (NPLP), subsequently merges with the medial cervical lymphatics, which ultimately connect to the deep cervical lymph nodes (85). Structurally, the plexus consists of a flattened network of unicellular capillaries characterised by button-like and zipper-like junctions, numerous unique valves, as well as the absence of a smooth muscle layer (85). These unique histological features allow the NPLP to be classified as a form of precollecting lymphatics, with extensive valvular structures to ensure the unidirectional flow of CSF towards the deep cervical lymph nodes.

**Figure 3:**
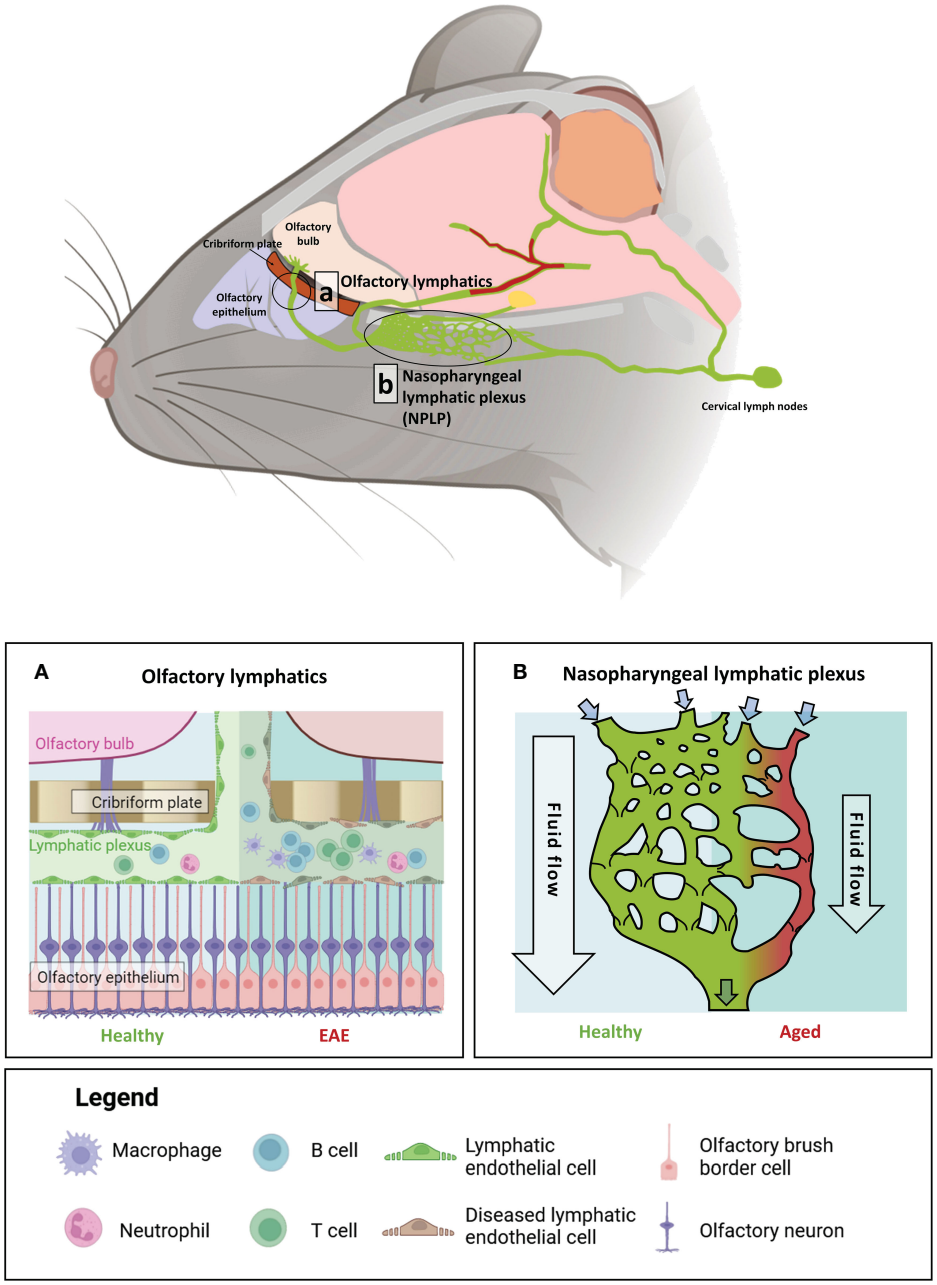
The impact of age and auto-immune insult on brain-border lymphatics in *Mus musculus*. Brain-border lymphatics are integral for the removal of metabolic waste and immune cells from the CSF. However, their function can diminish with advancing age or inflammatory insult. Top panel: a schematic of the mouse brain displaying the locations of the olfactory lymphatics **(A)** and nasopharyngeal lymphatic plexus (NPLP) **(B)**. **(A)** In healthy mice (left), lymphatics at the olfactory bulb drain CSF from the meninges and olfactory bulb. Mice with experimental autoimmune encephalomyelitis (EAE) display significant dilation of the lymphatic vessels with increased drainage of immune cells from the CSF (right). **(B)** The NPLP is a recently discovered anastomotic plexus found posterior to the olfactory lymphatics, present in both mice and humans. In healthy mice (left) the NPLP primarily serves to collect CSF that is drained from the ventral regions of the brain, before it is sent to the cervical lymphatics, which travel to deep cervical lymph nodes. During ageing (right), atrophy of the NPLP occurs, which can result in poorer drainage of lymphatic fluid that is likely to impair CSF flow. This age-related impaired lymphatic flow may contribute to the pathology of various neurodegenerative diseases. Figure created with BioRender.

In addition to its role in regulating CSF flow, emerging research suggests that the extensive nasal lymphatic microenvironment responds dynamically to inflammatory signals from the brain. In an induced EAE mouse model, significant lymphangiogenesis and dilation were observed at both the dorsal and ventral ends of the cribriform plate (88). Furthermore, CD11c-expressing cells and immune cells from the brain parenchyma were found to have drained at the cribriform plate in response to neuroinflammation (88). Notably, atrophy of the NPLP was observed in aged mice, hindering CSF flow and drainage, with transcriptomic profiling revealing elevated expression of genes involved in type I interferon signalling and the leukocyte response, such as *Il3*, *Mcl1* and MHC genes (85). These results suggest an involvement of lymphangiocytes, or the potential accumulation of immune cells within the nasal lymphatics, in modulating neuroimmune responses. Further research is required to characterise the immune profiles within this unique structure, and to determine whether it contains an active immune niche, like the DALTs, which can modulate immune responses in the brain. An overview of the brain-border lymphatics in mouse, alongside the impact of age and inflammatory insult on these processes, is shown in **Figure 3**.

## 3 Contribution of brain-border immune niches to neurodegenerative diseases

### 3.1 Alzheimer’s disease

AD is the most common neurodegenerative disease, observable by the formation of amyloid beta (Aβ) plaques and tau neurofibrillary tangles (NFTs). During early disease stages, neuronal atrophy is more significant within the temporal brain regions such as the hippocampus, resulting in dementia (89, 90). AD is thought to occur largely because of Aβ misfolding and tau hyperphosphorylation, which result in the accumulation of pathological Aβ plaques and tau NFTs. These are widely regarded as major contributors towards neuronal dysfunction and synaptic loss underlying neurodegeneration (91, 92). Additional hypotheses suggest a role for environmental factors, such as prolonged exposure to heavy metals, pesticides, and air pollution (93–96), in AD presentation, further complicating investigations into disease pathology. The exact underlying aetiology of sporadic AD remains unknown, and thus, current treatment options are limited. First-line treatments (donepezil, galantamine, rivastigmine and memantine) aim to treat cognitive symptoms by increasing cholinergic neurotransmission or, in the case of memantine, reducing glutamatergic neurotransmission to attenuate excitotoxicity. Recently, research has focused more on targeting the clearance of pathological Aβ, which is the mechanism of action of the FDA-approved drugs aducanumab and lecanemab. Although these improve on older treatments by targeting what is believed to be the main driver of pathology, as opposed to downstream effects on cognition, their use remains controversial due to their significant side effects and failure to halt disease progression (97, 98). This is likely due to the complex, multifactorial nature of AD.

Immune system interference has been observed in the AD brain since the disease was first described, when Alois Alzheimer noted reactive gliosis in patient autopsy samples (99–101). Since then, neuroinflammation has been regarded as a key manifestation associated with disease progression. Observations in human patient brain tissue and amyloid mouse models indicate that activated microglia colocalise with Aβ plaques, proposing an important role for glia in the clearance of Aβ from the brain (102–104). Variants of the *Trem2* gene, which encodes for the triggering receptor expressed on myeloid cells-2, have been strongly associated with the presentation of late-onset Alzheimer’s disease (LOAD) (105, 106). Studies using cultured neurons and *Trem2* knockout mice have suggested that the activation of TREM2 receptors promotes microglial phagocytosis and proliferation (107, 108), enabling the uptake and degradation of, and thus protection against, Aβ oligomers (109, 110). However, reactive microglia and astrocytes which interact with Aβ have been shown to release inflammatory cytokines, such as IL-1β, IL-6 and TNF-ɑ, across a number of experimental models, including primary mouse microglia culture and APP/PS1 mice, as well as in AD patient brain samples (111–113). These processes have been suggested to seed initial inflammation, triggering a cascade that results in the amplification of the neuroinflammatory response over time. This points to a possible fundamental role for the sustained immune response in the onset of AD (114). Therefore, the precise function of glia, including whether they are protective and the extent to which they contribute to disease progression, remains uncertain, making their targeting for therapeutic advances challenging.

Studies have suggested that dysregulation of the peripheral immune system, in addition to that of the CNS, may also contribute towards AD progression (101), due to the observation that AD patients present with significantly higher levels of peripheral pro-inflammatory cytokines than healthy subjects (115, 116). However, studies that attempt to link peripheral cytokine levels with disease severity in AD patients have so far yielded mixed results (117, 118). Elucidating mechanisms that underlie crosstalk between peripheral and central immune processes throughout disease progression, and specifically the role played by brain-border immune niches in neuroinflammation and pathology progression, is therefore of significant research interest. The presence of Aβ in human cervical lymph nodes suggests that it is cleared via the glymphatic system (119). In mice, CD163- and LYVE1-expressing macrophages in perivascular spaces have been reported to regulate arterial motion which drives the flow of CSF, thereby influencing the rate of Aβ clearance from the brain (70). The glymphatic system is most active during sleep, and its declining function with age has been linked with sleep disturbances and neurodegenerative disease progression (120). The brain-border immune niches and glymphatic system may therefore play an important role in the propagation of late-stage inflammation observed in AD. The increased presence of Aβ aggregates within meningeal vessels and the choroid plexus has been evidenced in AD patients (121), indicating that age-related impairments in meningeal lymphatic vessel drainage may be a key underlying mechanism of Aβ accumulation (122, 123). In addition, an increased number of neutrophils has been found to adhere to the choroid plexus and vasculatures and infiltrate into the hippocampus and cortex of patients (124). Furthermore, in mouse models of AD, neutrophil adhesion to endothelial cells at perivascular spaces has been shown to impair blood flow, resulting in diminished memory function (125). Deficiencies in meningeal lymphatics associated with aging are therefore highly likely to amplify Aβ aggregation in both the meninges and parenchyma, contributing to cognitive dysfunction in AD (126, 127).

Recent studies have identified a potential role for the CBM in enhancing the progression of AD. During neuroinflammation in mice, monocytes and neutrophils from the bone marrow are recruited into the meninges through small osseous channels. A similar role for these channels in humans has been suggested, although this has yet to be confirmed (74, 76, 128). Positron emission tomography (PET) imaging using radioligands for translocator protein (TSPO) signal has revealed significant inflammation, specifically within the frontal and parietal regions of the CBM, in AD patients (73). In the calvaria, TSPO readings were positively correlated with decreased Aβ42 concentration in the CSF, but not decreased Aβ40 concentration. This is particularly significant when considering the specific role of Aβ42 in AD pathogenesis. Aβ40 and Aβ42 are products of the differential cleavage of Aβ, with 40 and 42 amino acid residues, respectively. Despite being derived from the same precursor, these isoforms possess significant differences in their physicochemical and biological properties. In particular, the Aβ42 isoform is more prone to aggregation, and thus to the formation of Aβ plaques, and is more neurotoxic than Aβ40 (129, 130). As a result, Aβ42 is generally considered to play a greater role in AD pathology. Consequently, the observed correlation between increased TSPO readings in the calvaria and decreased Aβ42 concentration in the CSF suggests a potential link between cranial inflammation and AD pathology, which may contribute towards increased fibrillar Aβ42 deposits in the brain (73). Given these insights it is likely that a dual approach for targeting both immune niches and underlying amyloid plaques may be beneficial for the treatment of AD.

### 3.2 Parkinson’s disease

Like AD, PD is a progressive neurodegenerative disease that is caused by aberrant aggregates of misfolded proteins within the brain. PD is characterised by physical symptoms such as uncontrolled shaking, stiffness, bradykinesia, and a loss of balance. The disease can also present with several psychological symptoms including sleep problems, anxiety and depression and cognitive impairment (131). The main pathological hallmark of PD is the presence of Lewy Body formations, which begins in the substantia nigra of the midbrain and causes dopaminergic neuronal death (132). As the disease progresses, neurodegeneration and tissue damage spread to the rest of the brain. The formation of Lewy bodies is attributed to the misfolding of alpha-synuclein (ɑ-synuclein), resulting in the accumulation of synuclein fibrils (133). The reason for this occurrence in patients with sporadic PD is unclear, but evidence suggests it is likely due to complex interplay between genetic and environmental factors. Multiple gene variants have been linked with the misfolding of ɑ-synuclein and presentation of sporadic PD (134–136), whilst studies have suggested that overexposure to environmental toxins (137) and pesticides (138) could lead to gut microbiota changes that increase PD susceptibility (139). Disease presentation has also been associated with levels of exercise (140), caffeine intake, smoking (141) and traumatic brain injury (142). As with AD, the apparent multifactorial nature of PD makes it extremely difficult to treat effectively. Drugs currently approved for PD treatment include levodopa and dopamine agonists to increase dopaminergic transmission, and monoamine oxidase-B inhibitors to reduce the breakdown of dopamine in the synapse, thereby increasing binding to receptors. Although drugs targeting dopaminergic neurotransmission are initially effective at improving motor symptoms that manifest early in PD presentation, they are known to lose therapeutic efficacy as the disease progresses (143). No drugs currently exist to address the underlying pathology of PD to halt disease advancement.

Multiple inflammation-related genes have been identified as risk factors in the presentation of PD (144), and as such neuroinflammation has been suggested to exacerbate its progression (145, 146). The role of the inflammatory response in PD has been studied since observations that postmortem patient samples contain reactive microglia expressing MHC-II cell surface receptors (147). This leads to the excessive release of proinflammatory cytokines in the striatum, ventricular CSF and spinal CSF (148). The lentiviral-mediated overexpression of ɑ-synuclein in mouse microglia has been reported to result in neurodegeneration of dopaminergic neurons before the aggregation of endogenous ɑ-synuclein, suggesting that microglial activation may be a primary driver of disease pathology in the CNS (149). As well as dopaminergic degeneration, the accumulation of ɑ-synuclein in microglia also leads to the excessive release of pro-inflammatory and oxidative molecules. A proposed mechanism for microglial activation by ɑ-synuclein, characterised in microglial cell lines, primary cultured cells, and mouse models, depends on the binding of the protein to microglial CD11b. This activates NADPH oxidase (NOX2) via the initiation of RhoA pathway signalling (150). The activation of NOX2 increases the production of hydrogen peroxide (H_2_O_2_), which diffuses into the cytoplasm and directs microglial migration via the activation of Lyn, a tyrosine protein kinase (151). If this mechanism also occurs in human patients, the interaction of ɑ-synuclein with microglia, as opposed to its accumulation in dopaminergic neurons, may be a key component of the initial immune response driving degeneration of the dopaminergic system in PD. The exacerbation of the immune response and excessive release of pro-inflammatory molecules leads to further activation of microglia, in turn contributing towards the increased accumulation of ɑ-synuclein and dopaminergic degeneration in a feedforward cycle of neurodegeneration and inflammation (149, 152).

Braak’s hypothesis of PD suggests that the disease arises because of the presence of a pathogen in the gut and nasal cavity, before spreading towards and within the CNS (153–156). Mechanisms of neuroimmune crosstalk and the prevention of PD progression from the periphery into the brain are therefore essential components of research into improved therapeutic outcomes. The progression of the peripheral immune response into the brain during PD occurs due to the damaged integrity of the BBB, which leads to its increased permeability. This may be induced by ɑ-synuclein facilitated by astrocytic signalling (157, 158), or as a result of peripheral inflammation (159). A recent study has shown that the infiltration of lymphocytes into the brain parenchyma is mediated by BAMs residing in the choroid plexus and meninges, indicating a role for brain-border immune niches in facilitating neuroinflammation (14). In mice overexpressing ɑ-synuclein, BAMs were also observed to interact with ɑ-synuclein fibrils, present MHC-II complexes, and to colocalise with CD4^+^ T cells in the perivascular spaces. These processes have been suggested to initiate T cell antigen recruitment and parenchymal entry, providing a possible mechanism for immune cell entry into the brain during the early stages of PD that precedes neurodegeneration facilitated by ɑ-synuclein. Critically, this study also reported the presence of BAMs in close proximity to T cells in postmortem PD brain samples, suggesting that processes occurring in perivascular spaces are consistent in both animal models of the disease and human patients. Targeting these regions to prevent central neuroinflammation may therefore be a promising route of therapeutic intervention for PD.

Recently, diffusion tensor imaging (DTI) analysis in human PD patients has provided strong evidence that glymphatic system dysfunction occurs with PD progression (160, 161), and reduced meningeal lymphatic vessel flow has also been observed in idiopathic PD patients via MRI (162). Studies have looked to explore whether these changes to the glymphatic system amplify PD pathology. The accumulation of ɑ-synuclein in human patients has been shown to result in delayed lymphatic drainage, inflammation of the meninges and a reduced concentration of tight junctions between endothelial cells in the meningeal lymphatics system (162). In mice overexpressing ɑ-synuclein, the blocking of meningeal lymphatic drainage indeed exacerbated ɑ-synuclein accumulation, inflammation, and dopaminergic neurodegeneration, which heightened motor deficits (163). These studies highlight a potentially crucial role for immune niches in both the accumulation and clearance of ɑ-synuclein. Therefore, targeting these sites during early-stage PD may be useful in preventing the infiltration of pathology into the brain, whilst similar intervention during the later stages could be harnessed to slow disease progression by increasing the clearance of pathological ɑ-synuclein.

### 3.3 Multiple sclerosis

MS is a progressive autoimmune demyelinating disorder affecting approximately 36 per 100,000 people (164). Symptoms include extreme fatigue, sensory and visual disturbances, ataxia, and respiratory dysfunction (165). Initial diagnosis of MS is complex, and often requires a thorough examination of the patient’s family history, neurological exams, evoked potential tests, lumbar punctures, and MRI for focal white matter lesions, as according to the McDonald criteria (166). There are currently no ways to prevent the progression of the disorder, and patients are expected to relapse without warning. Corticosteroids are acute treatments that hasten recovery from relapse, but long-term corticosteroid treatment does not prevent further relapse (167), prompting the need for better therapeutic alternatives. Typically, MS can be classified into four categories depending on the manner of disease progression: clinically isolated syndrome (CIS), which is diagnosed when patients first experience neurological symptoms for over 24 hours; relapsing-remitting MS (RRMS), the most common diagnosis of MS, characterised by alternating periods of active and inactive disease progression; and the more active, aggressive primary progressive MS (PPMS) and secondary progressive MS (SPMS) subtypes.

The precise cause of MS is not known. However, it is believed that polymorphisms within immune-related genes (168, 169), and genes affecting myelin susceptibility to inflammatory insult (170), along with environmental factors, may cohesively contribute to aberrant lymphocyte activation underlying MS pathology (171, 172). A considerable proportion of MS-associated gene polymorphisms are found within the human leukocyte antigen (HLA-DR2) clusters, which reside within the highly polymorphic MHC-II region. Other reported genes with reported risk alleles include those encoding for interleukin receptor subunits, such as IL2RA and IL7RA (173, 174). Demyelination has also been associated with macrophage and B cell activity, reactive gliosis, altered oligodendrocyte progenitor cell (OPC) recruitment and axonal damage (175).

Compelling evidence suggests that immune cells in the choroid plexus play a role in the early pathogenesis of MS. In EAE mice, the number of CD4^+^ T cells increases in the choroid plexus and remains elevated throughout disease progression (176). Paracellular diapedesis of CD4^+^ T-helper 17 (Th17) cells into the brain parenchyma appears particularly crucial in the progression of MS pathogenesis (177), and the infiltration of Th17 cells has been shown to occur from the choroid plexus specifically. The expression of the chemokine receptor CCR6 on Th17 cells is necessary for adherence to CCL20^+^ choroid plexus epithelial cells, allowing T cells to pass into the CSF (178). This process appears to be facilitated by adenosine signalling; knockdown of adenosine A_2A_ receptors in the choroid plexus has been shown to attenuate diapedesis via inhibition of the NFκB/STAT3 pathway, leading to reduced CCL20 expression in the brain parenchyma (179). However, the diapedesis of effector Th cells (including Th17 cells) *in vitro* appears to be independent of CCR6-CCL20 signalling, suggesting that there might be alternative interactions at play (176). For instance, IFN-γR1 expressed within the choroid plexus has been shown to reduce the local expression of adhesion molecules and chemokines, preventing Th17 cells from infiltrating into the CNS (180). Given the incomplete understanding of this niche, further research is required to identify the precise mechanism facilitating Th17 cell entry into the brain during the pathogenesis of MS.

In addition to the choroid plexus, recent evidence highlights the significance of immune activity within the meninges in the pathogenesis of MS. Lymphoid-like follicular structures, predominantly composed of autoreactive B cells, are frequently observed adjacent to subpial demyelinating cortical lesions within the subarachnoid space of SPMS and PPMS patients (181). These follicular structures tend to be concentrated around the deep cortical sulci (181). Meningeal APCs have been identified as activators of CD4^+^ T cells within these follicles (182). Furthermore, the depletion of microglia and meningeal macrophages has been shown to reduce MHC-II and CD80 co-stimulatory molecule expressions, diminishing T cell reactivation and proliferation, and consequently halt demyelination events in EAE mice (183), implicating immune cell activity at the meninges in MS pathology.

It has been suggested that glymphatic flow becomes impaired at the perivascular spaces in MS patients. Dilation of the perivascular spaces is observed in some MS patients, although its correlation to severity of MS and the disease progression remains uncertain (184). In a recent study using DTI to analyse fluid diffusion along the perivascular spaces, a negative association between diffusivity index (a proxy for glymphatic function) and disease duration was observed at the onset of disease course, suggesting an early impairment of glymphatic clearance in MS patients (185). Another study has suggested that metabolic dysfunction in perivascular astrocytes may result in the diffused expression of AQP4 in astrocytic end feet, and its expression at lesion sites has been found to decrease in response to immune elements (186). AQP4 is reported to be an integral component of the glymphatic system and so its reduced expression is likely to lead to alterations in glymphatic clearance (187). However, it is currently unknown if impairment in glymphatic flow and perivascular space dilation are causal factors in the pathology of MS, or consequences arising from a physiological response to MS lesions. Consequently, further investigation is required to determine how glymphatic flow dysfunction contributes to MS pathology.

The CBM may be another brain-border niche that contributes to MS pathogenesis. Indeed, PET scans using radioligands for TPSO have revealed that inflammatory activity tends to be heightened at the skull base in both EAE mice and MS patients (73). This could potentially be explained by autoreactive CXCR4^+^ myelin-reactive T cells migrating into the bone marrow through ossified skull meningeal channels to augment myelopoiesis through the CCL5-CCR5 axis (78). However, it is not currently known why the skull base specifically is activated, and direct associations between myeloid cells generated from the CBM and the lymphatics also remain to be investigated.

**Figure 4:**
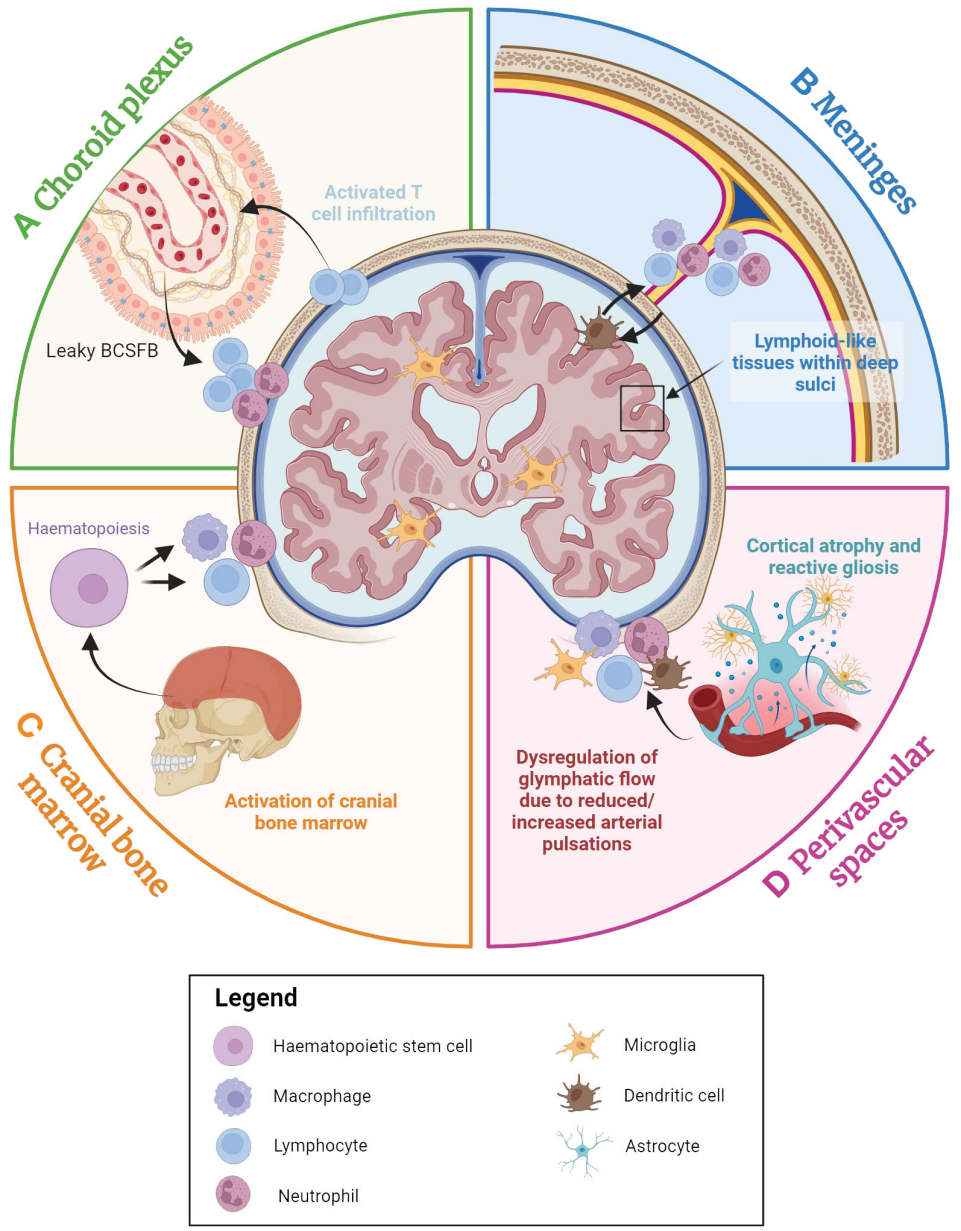
An overview of the contributions of the brain-border niches to neurodegenerative diseases. The choroid plexus, cranial bone marrow (CBM), perivascular spaces and meninges all appear to play a role in neurodegenerative disease. **(A)** The choroid plexus serves as the primary brain-cerebrospinal fluid barrier (BCSFB) and actively responds to immune factors from both the CSF and the blood stream. With advancing age and during the inflammatory response, immune cell populations accumulate within the choroid plexus, and the BCSFB becomes leakier due to the loss of tight junctions, potentially facilitating greater peripheral immune cell activation and infiltration into the brain. **(B)** In many age-associated neurodegenerative diseases, high concentrations of inflammatory factors appear to result in the accumulation of myeloid and lymphoid cells in the dura, subarachnoid space, and even within the deep sulci of the cortex, which may perpetuate neurodegeneration. **(C)** The CBM is a brain-border niche containing haematopoietic stem cells, which can differentiate to myeloid and lymphoid cells in response to a range of immune factors. Evidence suggests that immune activation in the CBM is associated with various neurodegenerative diseases. **(D)** Cortical atrophy and gliosis associated with neurodegenerative disease may lead to a leaky blood-brain barrier (BBB), while inflammatory factors from the parenchyma may induce increased immune cell localisation in perivascular spaces. Furthermore, dysregulation of glymphatic flow due to factors such as diminishing arterial pulsations may affect clearance of metabolic waste and immune cells within the brain parenchyma, leading to the build-up of neurotoxic molecules within the brain. Figure created with Biorender.

Evidence has also implicated structural changes to the lymphatic system in the pathology of MS. A recent study has reported the occurrence of profound lymphangiogenesis in EAE mice following the proliferation of lymphatic endothelial cells at the nasal lymphatics near the cribriform plate (88). In this study, the lymphatic endothelial cells were found to have proliferated as a response to inflammation, and this proliferation has been shown to depend on vascular endothelial growth factor (VEGF) C signalling (88, 188, 189), although an alternative mechanism for this process has been proposed that requires the transdifferentiation of activated monocytes into lymphatic endothelial cells (190, 191). These findings demonstrate the occurrence of lymphatic vessel remodelling in EAE mice, suggesting a potential role for this process in MS pathology. Indeed, it is feasible that lymphangiogenesis in proximity to the brain could facilitate greater immune cell infiltration into the brain; a known contributor to MS progression (178, 192, 193). Other studies have found evidence that immune activation at specific lymph nodes may also underlie aspects of MS pathology. Lymph nodes are known as ‘collecting centres’ where APCs come into close contact for priming T cells (194). The medial and lateral cervical lymph nodes receive lymphatic fluids from the brain parenchyma and meninges, and activation of T cells in these regions may contribute to humoral activation during the early stages of MS pathogenesis (195). Accordingly, activated cells from the cervical lymph nodes may re-enter the brain through blood circulation into the dura, perivascular spaces, or choroid plexus. Here they interact with or secrete factors such as IFN-γ to prompt secondary responses within these niches, promoting disease progression (195). Excision of cervical lymph nodes (196) and high-intensity focused ultrasound in cervical lymph nodes for lymphocyte ablation (197), have both been shown to significantly reduce relapse severity in EAE mice, suggesting that this putative disease pathway may be a valid therapeutic target. The contributions of brain-border immune niches to the presentation of neurodegenerative diseases are highlighted in **Figure 4**.

## 4 Considerations in the study and clinical use of brain-border immune niches in neurodegenerative diseases

Neurodegenerative diseases are progressive disorders that can be classified into stages depending on the severity of symptoms and histological profiles. While current therapies hold some promise for slowing disease advancement and improving symptoms in initial stages, they have proved less effective for patients diagnosed in intermediate and advanced stages. Moreover, the aetiologies of these diseases remain elusive, meaning there are limited therapies for preventing or curing them. In AD and PD, neurodegeneration is putatively caused by the introduction of misfolded proteins within the CNS that undergo uncontrolled prion-like propagation and aberrant aggregation, triggering immune responses (198). Microglia and astrocytes form a first-line defence against aggregates. However, this initial neuroinflammatory response from within the parenchyma may trigger a chain reaction in wider brain-border niches, which may contribute to disease progression. Therefore, it is possible that targeting these immune niches could prevent a secondary immune response and slow disease progression in the case of AD and PD. For MS, immunosuppression appears effective as a disease-modifying therapy (DMT), particularly for RRMS and SPMS (199). However, the precise mechanisms and aetiology of MS remain unknown, and treatments are therefore limited, particularly in the case of PPMS. As explained previously, recent findings report the accumulation of immune cells at the brain-borders in MS, which may later infiltrate and release immune factors into the CNS parenchyma (178, 179, 192, 193). Therefore, controlling the immune buildup at the brain-borders may be an efficient acute therapy for MS.

Whilst current literature exploring the brain-border immune system has provided valuable insights, the lack of tools available to isolate the specific role of each brain-associated immune niche in any given neurodegenerative disease poses a significant challenge when attempting to understand their roles in the aetiologies of these conditions. Moreover, although the targeting of immune niches for the treatment of neurological diseases may seem like a promising therapeutic approach, a similar lack of tools to deliver isolated treatment delivery into specific immune niches makes this difficult to achieve at present. Consequently, it is important to consider caveats of current approaches to explore the roles of these immune niches in health and disease, and how we may address these limitations in future investigation and clinical application.

### 4.1 Challenges associated with the study of immune niches in neurodegenerative disease

To effectively target brain-border immune niches in the treatment of neurodegenerative disease, it is important to understand how these function in health. However, studying these niches in humans is fraught with ethical limitations. Due to the highly invasive nature of biopsies required to access the brain and its bordering regions, investigation of these regions in humans is typically limited to donated post-mortem samples (200, 201). Although valuable, these likely do not represent a true cross-section of healthy tissues across the population, as it is possible that the manner of death may affect the cellular/molecular profile of samples. Similarly, post-mortem diseased tissue is typically only available from late-stage or terminal disease donors, neglecting the dynamical nature of neurodegenerative diseases and the range of immune states they inhabit during their progression. The relatively small sample sizes this issue produces limits the power of findings derived from post-mortem samples. These studies also require strict measures to be undertaken to ensure optimal preservation of DNA, RNA, and protein contained within the brain. However, this can be difficult to achieve due to pre-mortem events, such as hypoxia, and post-mortem delays (202). Because of these difficulties regarding access to and preservation of human tissue, a large proportion of studies in this field rely on animal models, in which tissue is more readily available and experimental conditions are less constrained. However, these studies also come with significant drawbacks. The most apparent limitation of animal models is the differences in neurobiology between model species and humans (203–206). Indeed, rodents, the primary models in this field, lack the full complement of glial complexity seen in humans, and certain vascular and immunological components are also absent (207). Moreover, their short lifespans limit the development of progressive diseases (208), potentially leading to the incomplete development of molecular and cellular pathological hallmarks in brain-associated immune niches. Perhaps because of these reasons, rodent models of neurodegenerative disease are generally considered poor in terms of their predictive validity (209). To utilise these models effectively in the study of immune niche involvement in neurodegenerative disease, it may be valuable to attempt further reverse-translational studies using non-invasive neuroimaging methods, that are currently used in humans, in rodent models (in which high fidelity data is readily available for comparison) to validate their use in humans.

### 4.2 Monitoring the activity of immune niches through functional and structural imaging

Imaging modalities used for the formulation of diagnoses and prognoses of neurological diseases can provide detailed information about structural or physiological brain changes; due to their non-invasive nature, they are among the most utilised tools to conduct clinical studies and evaluate neurological disease progression. Anatomical imaging techniques such as computed tomography (CT) or MRI may be combined with functional imaging using PET or DTI, respectively, for acute visualisation of aberrant physiological changes in patients. For example, MRI coupled with DTI along the perivascular space (DTI-APS) showed that decreased diffusivity index correlated with increased perivascular burden in both AD and PD patients (161, 210), suggestive of reduced glymphatic flow. On the other hand, the use of CT combined with PET tracers specific to cells of the myeloid lineage is currently being investigated in preclinical studies concerning AD and MS. Examples of promising PET radiotracers include those binding to TSPO (73, 211, 212), GPR84 (213), and triggering receptor expressed on myeloid cells 1 (TREM1) receptor (214), while lymphoid tracers include nanoparticle conjugated CD-19 monoclonal antibodies for B cells (215) and FAraG for T cells in EAE models (216). However, despite their utility, these techniques are not without their limitations. MRI/CT and PET lack the spatial resolution to precisely measure single-cell changes and interactions within these niches triggered by neuroinflammation (217, 218). This makes it challenging to attribute inflammatory functions to specific cell types and their interactions within immune niches, which likely form an important part of the aetiology of neurodegenerative diseases. Furthermore, whilst PET scanning may be able to provide valuable information about specific disease-relevant cell populations or biomarkers over larger areas, the utility of the tracers used is limited to our understanding of their biology. For example, TSPO, which is one of the most widely utilised PET markers in the study of neuroinflammation, is known to be upregulated by astrocytes and microglia upon their activation and by infiltrating macrophages (219), however, its sources from the periphery are less well understood. This brings into question the source and specificity of TSPO signal in the CNS, in studies using radioligands for this marker (220, 221). Consequently, the lack of specificity and our understanding of the origins of these markers limits our ability to understand the roles and interactions of immune-related molecules or immune cell types in these niches. However, it is expected that with further development of neuroimaging techniques and increased understanding of relevant molecular biomarkers, studies in humans will soon provide more reliable data as to the cellular and molecular makeup of these niches in both disease and health, facilitating their use in disease diagnosis, progression, and clinical study.

### 4.3 Promoting glymphatic flow and metabolic waste clearance

The obstruction of glymphatic pathways is a common hallmark of neurodegenerative disease that results in failure to clear metabolic waste, thus leading to the accumulation of immune cells within the perivascular spaces. Therefore, it is hypothesised that increasing glymphatic clearance may be a promising therapeutic approach, as it would allow the removal of aggregates and toxic metabolites from the brain parenchyma.

Studies have shown that stiffening of the arteries may lead to impedance of glymphatic flow in a hypertensive rat model (222). Controlling hypertension may therefore be an effective method for treating individuals with glymphatic obstruction associated with neurodegenerative disease, particularly in age-associated dementia, as age is positively correlated with arterial stiffness (223). Non-pharmacological interventions to control hypertension involve lifestyle changes such as weight loss, increased cardiovascular exercise, dietary changes, and reduced salt intake (224). Pharmacological options for treating hypertension include diuretics, angiotensin-II-receptor antagonists (sartans), beta-blockers, and calcium-channel blockers (225).

Current studies have also revealed several other promising agents that may aid in improving glymphatic flow. VEGFC is a lymphangiogenic factor that has been shown to improve glymphatic drainage (126, 226), and therefore potentially enable the clearance of Aβ from the brain parenchyma. Vasoconstrictors, such as α_2_-adrenergic agonists, may be used to dilate the glymphatic channels for intrathecal lumbar administration of medications (227), allowing direct delivery of biologics or drugs to the CNS, which may otherwise not be able to cross the BBB or BCSFB.

In addition to using drugs or biologics, clearing obstructed glymphatic channels has also been achieved in animal models using mechanical methods such as focused ultrasound treatment in combination with microbubbles (FUS-MB) (122), and non-invasive neuronal stimulation techniques, such as transcranial magnetic stimulation (TMS) or multisensory stimulation (228, 229). A recent study using the 5xFAD AD mouse model showed that treatment with FUS-MB led to enhanced solute Aβ clearance from the brain, first into the CSF space and then into the deep cervical lymph nodes, which correlated with improved memory functions (122). These findings suggest that non-pharmacological treatment methods, such as transcranial magnetic stimulations at clinics, coupled with multisensory interventions at home, may also be viable therapeutic approaches to promote impaired glymphatic clearance and thus improve disease symptomology in AD patients.

### 4.4 Preventing immune cell infiltration into the brain parenchyma

The infiltration of immune cells into the brain parenchyma is a significant event associated with neuronal atrophy in numerous neurodegenerative disorders (230, 231). Once within the brain parenchyma, activated lymphocytes release inflammatory cytokines that impair neuronal function, whilst invading myeloid cells are also known to release various cytotoxic and neuroinflammatory factors. Consequently, the specific mechanisms that modulate immune cell invasion into, or egress out of, the brain parenchyma hold promise as potential therapeutic targets.

Neutrophil migration into the brain parenchyma has been observed using PET scans in transgenic AD models (232). Neutrophil depletion or the inhibition of neutrophil trafficking via lymphocyte function-associated antigen 1 (LFA-1) blockade has been shown to reduce AD–like neuropathology and improve memory in mice already showing cognitive dysfunction (124). This indicates that the prevention of neutrophil trafficking to the brain parenchyma may be a valid therapeutic approach for the treatment of AD. Similar approaches, for example, the modulation of α4-integrin-mediated trafficking, have shown promise in the treatment of amyotrophic lateral sclerosis (ALS). α4-integrin is a heterodimeric cell surface marker for leukocytes and is reported to be important in facilitating the migration of leukocytes into the brain parenchyma after neural inflammation or injury (233). Studies have shown that intraperitoneal injection of natalizumab, an anti-α4-integrin monoclonal antibody, is able to block the infiltration of T cells and natural killer cells into the CNS of an ALS mouse model, effectively preventing inflammation and cytokine release in the brain parenchyma and preserving motor function (231). Ultimately, more research is needed to determine the potential of immune-trafficking-modulating therapies in the treatment of human neurodegenerative disease, but initial findings are promising.

### 4.5 Modulating cranial bone marrow-derived cell populations

In the context of treating neurodegenerative disorders, there have been several significant findings regarding immune activity at the CBM. For example, polymorphonuclear Ly6G^+^ monocytes and neutrophils derived from the CBM have been observed to modulate the function of other adaptive immune cells, thereby exhibiting an immunoregulatory role, in contrast to infiltrating Ly6G^+^ cells derived from the blood, which have been reported to display a more inflammatory phenotype (76). These findings therefore provide evidence for the existence, and thus potential manipulation, of immunoregulatory cells from the CBM for therapeutic purposes. What is more, in mice recovering from EAE, Ly6G^+^ cells have been observed to become recruited to the meninges, where they are converted to myeloid-derived suppressor cells (MDSCs), ultimately suppressing CD138^+^ B cell accumulation in the meninges (234). This recruitment and conversion process points to the existence of an endogenous mechanism mitigating neuroinflammation in the context of neurodegenerative disease. Indeed, Ly6G^+^ cells are known to be converted to MDSCs through the activation of the STAT3-dependent signalling pathway (234), therefore providing a precise molecular target for therapeutic intervention in this process. Furthermore, compounds such as cannabidiol and IFN-β have been shown to promote the localisation of MDSCs in the meninges (234), and improve their suppressive functions (235). Furthermore, transcranial application of CXCR4 antagonist AMD3100 into the CBM has been shown to facilitate the migration of Ly6G^+^ cells into the dura mater (76), indicating that manipulation of MDSCs at the CBM may be a viable strategy for mitigating neuroinflammation in neurodegenerative disorders.

However, specifically modulating CBM-derived cells effectively and precisely may prove to be challenging due to the technical difficulty of accessing the CBM and isolating and targeting specific cell types or molecular targets within this niche. Additionally, the exact routes by which CBM-derived cells are given access to the brain parenchyma have not been fully elucidated, meaning it is unclear how effective the delivery therapeutics via this channel would be (or to precisely where in the brain they would be delivered). Osseous channels have been suggested to display heterogenous plasticity throughout life in a region-dependent manner, although their expression and how this is changed during ageing and neurological disease presentation has not been well-characterised (128). A more comprehensive understanding of these mechanisms is essential to facilitate therapeutic advances associated with targeting of the CBM.

### 4.6 Directed and intranasal delivery of drugs and biologics

Injections of drugs into the choroid plexus and ventricular system to allow delivery into the brain parenchyma is a promising way of bypassing the BBB, although therapeutic effects are reported to be limited by diffusion distance, particularly during the targeting of deeper tissue regions (236, 237). Studies have suggested that directed drug delivery into the meninges may exert more widespread effects to brain parenchymal regions (123). However, these findings have so far been restricted to rodent models, and it remains to be seen whether their impact will be as effective in human brain tissue (238). Additionally, the way in which CSF flow is disrupted due to neurodegenerative disease is not comprehensively understood, and factors that influence CSF flow have not been fully described (237), therefore limiting the advancement of therapeutics associated with drug delivery into the choroid plexus and meninges.

In recent years, there has been a growing interest in intranasal delivery, using agents such as adeno-associated viruses (AAVs) and nanoparticles, as an alternative, non-invasive method for administering therapeutics to the brain to address neurodegenerative diseases. Notably, the olfactory mucosa in the nasopharynx region serves as a highly accessible region for drug penetrance (85). This approach provides numerous advantages for brain-border-focused treatment: it is non-invasive, and the presence of highly vascularised lymphatic vessels in the nasal mucosa allows the administered agent to swiftly enter the lymphatic system. Additionally, intranasal administration reduces first-pass metabolism at the liver compared to intravenous delivery. Agents administered intranasally can directly affect the lymphatic endothelium and olfactory bulb by modulating the release of inflammatory cytokines (239). Moreover, agents can travel through the lymphatics to reach the cervical lymph nodes, potentially targeting immune cells for gene therapy and precise immunotherapy. In addition to targeting brain-border niches, intranasal applications offer the benefits of either direct or indirect delivery into the brain. Preclinical studies in mice have shown that combining EGFP-AAV delivery with methods like FUS-MB can effectively target specific brain regions with higher efficacy compared to similar treatments via intravenous injections (240, 241).

However, the effectiveness of intranasal delivery is hindered by the rapid clearance of nasal passages due to mucociliary movement, which reduces the bioavailability of administered treatments. To circumvent this limitation, efforts have been made to coat particles with mucoadhesive substances and/or to co-administer enzymes, thus improving drug bioavailability (242, 243). Further research is required to develop novel nanocarriers for improved intranasal drug delivery, whilst ensuring that unintended adaptive responses are not provoked by such techniques.

## 5 Conclusion

Over the last century, understanding of the barriers surrounding the brain has advanced considerably, from initial beliefs that the brain was encompassed by an absolute, impermeable barrier, to more recent studies revealing the presence of brain-border immune niches. Crucially, modern-day studies have demonstrated a much greater degree of immune communication between the periphery and CNS than was previously believed. Indeed, peripheral immune cells that accumulate in brain-border regions appear to play an important role in neuroinflammatory processes via the facilitation of immune cell entry into the CNS. This has been reported to occur in several brain-border immune niches, including the choroid plexus, the meninges, and the perivascular spaces. Furthermore, the CBM is also the subject of considerable interest due to the discovery that its immune cells and immature progenitors can enter the brain via ossified channels, whilst the nasal lymphatic system contributes to the control of immune cell drainage from the brain parenchyma. With these findings in mind, it is evident that brain-border immune niches collectively play a significant role in neuroinflammatory processes occurring in the CNS, as well as in the control of neuroimmune interactions between the brain and periphery.

Given these significant findings, research into brain-border immune niches appears to have the potential to advance our understanding of the pathology of numerous brain conditions, in particular neurodegenerative diseases, such as AD, PD and MS. These have all been reported to present with alterations to the inflammatory response in both the brain and periphery, and pathological proteins have been observed to accumulate in brain-border immune niches throughout disease progression. Therefore, activity at the borders of the brain has been proposed to facilitate the infiltration of immune cells into the brain, thereby driving the neuroinflammatory response and thus, contributing to disease progression. Consequently, developing a greater understanding of these niches using both animal models and clinical studies may have significant implications for the diagnosis, prognosis, and development of novel therapeutics for neurodegenerative diseases, which currently remain inadequate.

In the past, the development of drugs that can effectively target the CNS has proven notoriously difficult due to the relative impermeability of the BBB and BCSFB. However, with further research, this may soon change. In the next decade, further investigation into the brain-border niches is expected, with the potential to shed light on the heterogeneity of each niche at single-cell resolution or with spatial transcriptomics. These studies have the potential to reveal the cellular players and immune factors that contribute to the progression of these debilitative diseases, thereby leading to the discovery of specific immune-related biomarkers and potential therapeutic targets. Moreover, continuous advancements in imaging resolution and the development of novel PET radioligands are likely to enable earlier diagnosis of neurodegenerative diseases and be effective in determining disease classification and improving prognosis. Additionally, it is important to note that brain-border niches themselves provide opportunities for therapeutic intervention. Protection against disease-exacerbating neuroinflammation may be achieved by preventing immune cell infiltration from brain-border niches into the parenchyma, or by modulating the function of CBM-derived suppressor cells. Alternatively, evidence also suggests that promoting glymphatic flow may represent another therapeutic avenue, aiding in the removal of pathological proteins from the brain parenchyma and immune niches. Finally, the CBM and nasopharyngeal lymphatic plexus may act as alternative access routes for drugs to enter the brain, for example via intranasal delivery, and thus are beginning to emerge as less-invasive routes of delivery to the CNS for promising new therapeutics.

## Author contributions

LT: Writing – review & editing, Writing – original draft, Conceptualization. GC: Writing – review & editing, Writing – original draft, Conceptualization. MH: Writing – review & editing. XY: Writing – original draft, Conceptualization, Writing – review & editing. CO: Writing – review & editing. BJ: Writing – review & editing. JK: Writing – review & editing. JP: Writing – review & editing. M-SK: Writing – review & editing. MK: Writing – review & editing. SJ: Writing – review & editing, Writing – original draft, Supervision, Funding acquisition, Conceptualization.
